# Impact of a bi-annual community-directed treatment with ivermectin programme on the incidence of epilepsy in an onchocerciasis-endemic area of Mahenge, Tanzania: A population-based prospective study

**DOI:** 10.1371/journal.pntd.0011178

**Published:** 2023-06-28

**Authors:** Dan Bhwana, Luís-Jorge Amaral, Athanas Mhina, Paul Martine Hayuma, Filbert Francis, Joseph N. Siewe Fodjo, Bruno P. Mmbando, Robert Colebunders

**Affiliations:** 1 National Institute for Medical Research, Tanga, Tanzania; 2 Global Health Institute, University of Antwerp, Antwerp, Belgium; Federal University of Agriculture Abeokuta, NIGERIA

## Abstract

**Background:**

Community-directed treatment with ivermectin (CDTi) is used to eliminate onchocerciasis. However, despite 25 years of annual CDTi in Mahenge, Tanzania, the prevalence of onchocerciasis and onchocerciasis-associated epilepsy remained high in certain rural villages. Therefore, in 2019, bi-annual CDTi was introduced in the area. This study assessed the impact of the programme on the incidence of epilepsy in four villages.

**Methodology:**

Door-to-door epilepsy surveys were conducted prior to (2017/18) and after (2021) implementing a bi-annual CDTi program. All household members were screened for epilepsy symptoms using a validated questionnaire, and suspected cases were examined by a medical doctor to confirm/reject the diagnosis of epilepsy. The prevalence and annual incidence of epilepsy, including nodding syndrome, were calculated with 95% Wilson confidence intervals with continuity correction. The latter was also done for CDTi coverage in 2016 and 2021.

**Results:**

Precisely 5,444 and 6,598 persons were screened for epilepsy before and after implementing the intervention. The CDTi coverage of the overall population was 82.3% (95%CI: 81.3–83.2%) in 2021 and sustained in both distribution rounds (81.5% and 76.8%). The coverage was particularly high in children and teenagers aged 6 to 18 years (93.2%, 95%CI: 92.1–94.2%). The epilepsy prevalence remained similar: 3.3% (95%CI: 2.9–3.9%) in 2017/18 versus 3.1% (95%CI: 2.7–3.5%) in 2021. However, the incidence of epilepsy declined from 177.6 (95%CI: 121.2–258.5) in 2015–2017 and 2016–2018 to 45.5 (95%CI: 22.2–89.7) in 2019–2021 per 100,000 persons-years. The incidence of probable nodding syndrome varied from 18.4 (95%CI: 4.7–58.5) to 5.1 (95%CI: 0.3–32.8). None of the nine incidence cases of epilepsy for which information on ivermectin intake was available took ivermectin in the year they developed their first seizures.

**Conclusion:**

A bi-annual CDTi programme should be implemented in areas with high prevalence of onchocerciasis and epilepsy. High CDTi coverage among children is particularly important to prevent onchocerciasis-associated epilepsy.

## Author summary

Onchocerciasis is a neglected tropical disease associated with epilepsy, particularly in areas of high *Onchocerca volvulus* transmission. Onchocerciasis-associated epilepsy is characterised by seizures that start between the ages of five to 18 years. The tropical disease can be controlled through community-directed treatment with ivermectin (CDTi). Mahenge, in Tanzania, had a high prevalence of onchocerciasis and epilepsy despite more than 20 years of annual CDTi. Hence, the Tanzanian Neglected Tropical Diseases Control Programme has switched from annual to bi-annual CDTi since 2019. After this switch, the CDTi coverage increased and was sustained in both ivermectin rounds in 2021, and the number of new epilepsy cases decreased. The latter were persons who did not take ivermectin the year they had their first seizures. Hence, all ivermectin-eligible children at risk of onchocerciasis should take ivermectin at least annually. Overall, increasing the frequency and coverage of the CDTi programme should be considered in onchocerciasis-endemic areas with a high prevalence of epilepsy.

## 1. Introduction

Onchocerciasis is a skin and eye disease and is associated with epilepsy, known as onchocerciasis-associated epilepsy (OAE) [1]. Onchocerciasis is caused by the filariae *Onchocerca volvulus* and is transmitted through bites of *Simulium* blackflies [2]. Over 19 million people are estimated to be infected, mostly in sub-Saharan Africa [3]. The World Health Organization (WHO) targeted onchocerciasis for elimination through annual community-directed treatment with ivermectin (CDTi) [2]. Ivermectin is a microfilaricidal that kills the progeny of *O*. *volvulus* and temporarily sterilises the adult worms [4].

In 1938, Casis-Sacre found a high prevalence of epilepsy in the onchocerciasis-endemic states of Chiapas and Oaxaca in Mexico [5]. In 1965, Jilek-Aall described the first nodding syndrome (NS) cases in Mahenge, an onchocerciasis-endemic area in Tanzania with a high prevalence of epilepsy [6]. High prevalence and incidence of epilepsy have been observed in areas with inadequate onchocerciasis control, like South Sudan [7] and the Democratic Republic of Congo [8]. Similarly, in northern Uganda, cases of NS and other forms of OAE emerged in onchocerciasis-endemic areas where there was no ivermectin distribution [9]. After introducing annual CDTi, the number of new NS cases started to decrease; and when control was intensified to bi-annual CDTi with ground larviciding of the rivers, new cases of NS stopped emerging and the overall incidence of epilepsy declined [9]. Likewise, since the elimination of onchocerciasis in 2004 in Kabarole, western Uganda, the overall incidence of epilepsy has significantly reduced, especially among persons aged three to 18 years, and no new cases of NS have been reported [10].

In 1989, before ivermectin became available, a survey in 11 villages in the Mahenge area, Ulanga district in Tanzania, documented an overall epilepsy prevalence of 11.4 (95%CI: 9.9–13.1) per 1,000 people and incidence of 73.3 (95%CI: 61.2–87.9) per 100,000 person-years [11]. Annual CDTi has been implemented in Ulanga since 1997. However, coverage was inconsistent and insufficient (<80%) to eliminate onchocerciasis transmission in hyperendemic foci such as Mahenge [12, 13].

In 2017, despite 20 years of CDTi, a study conducted in rural Mahenge recorded a high prevalence of epilepsy of 35 per 1,000 people in Mdindo and Msogezi villages [14]. At the time, the prevalence of *O*. *volvulus* antibodies in the population of the two villages, tested by Ov16 rapid diagnostic test, was 38.4% in children aged six to 10 years and 76.5% (95%CI: 68.7–84.2%) in males aged 20 years and older (potentially hyperendemic for onchocerciasis before control) [14]. The overall incidence of epilepsy in the two villages was estimated at 120 (95%CI: 70–203) per 100,000 person-years. In 2018, a high prevalence of epilepsy was also observed in two other rural villages in Mahenge, Mzelezi (28.8 per 1,000 people) and Sali (36.6 per 1,000 people) [15]. Of the persons with epilepsy (PWE), 77.9% met the criteria for the clinical case definition of OAE [15].

Although the annual ivermectin therapeutic coverage reported by the Ulanga district has been above 65% between 2003 and 2015, the percentage of *Simulium damnosum s*.*l*. carrying infective L3 stage parasites was 0.57% (95%CI: 0.43%-0.74%) in 2018 [16]. This value is similar to the percentage found in the 1960s before any onchocerciasis control intervention, although the sensitivities of the methods used were different [16]. Due to the persistent transmission of onchocerciasis and evidence that annual CDTi would be insufficient to eliminate *O*. *volvulus* in former hyperendemic foci [16, 17], the Tanzanian Neglected Tropical Diseases Control Programme initiated bi-annually CDTi in this area in 2019. This study investigated the impact of increasing the frequency of the CDTi to biannual on the incidence of epilepsy, including NS, in the four previously mentioned rural villages in Mahenge.

## 2. Methodology

### 2.1 Ethics statement

The study was conducted according to the guidelines of the Declaration of Helsinki. Ethical approval was obtained from the Ethics committee of the National Institute for Medical Research, Tanzania (NIMR/HQ/R.8a/Vol.IX/2278) and the Ethics Committee of the Antwerp University Hospital, Belgium (B300201837863).

### 2.2 Study site and population

**Fig 1:**
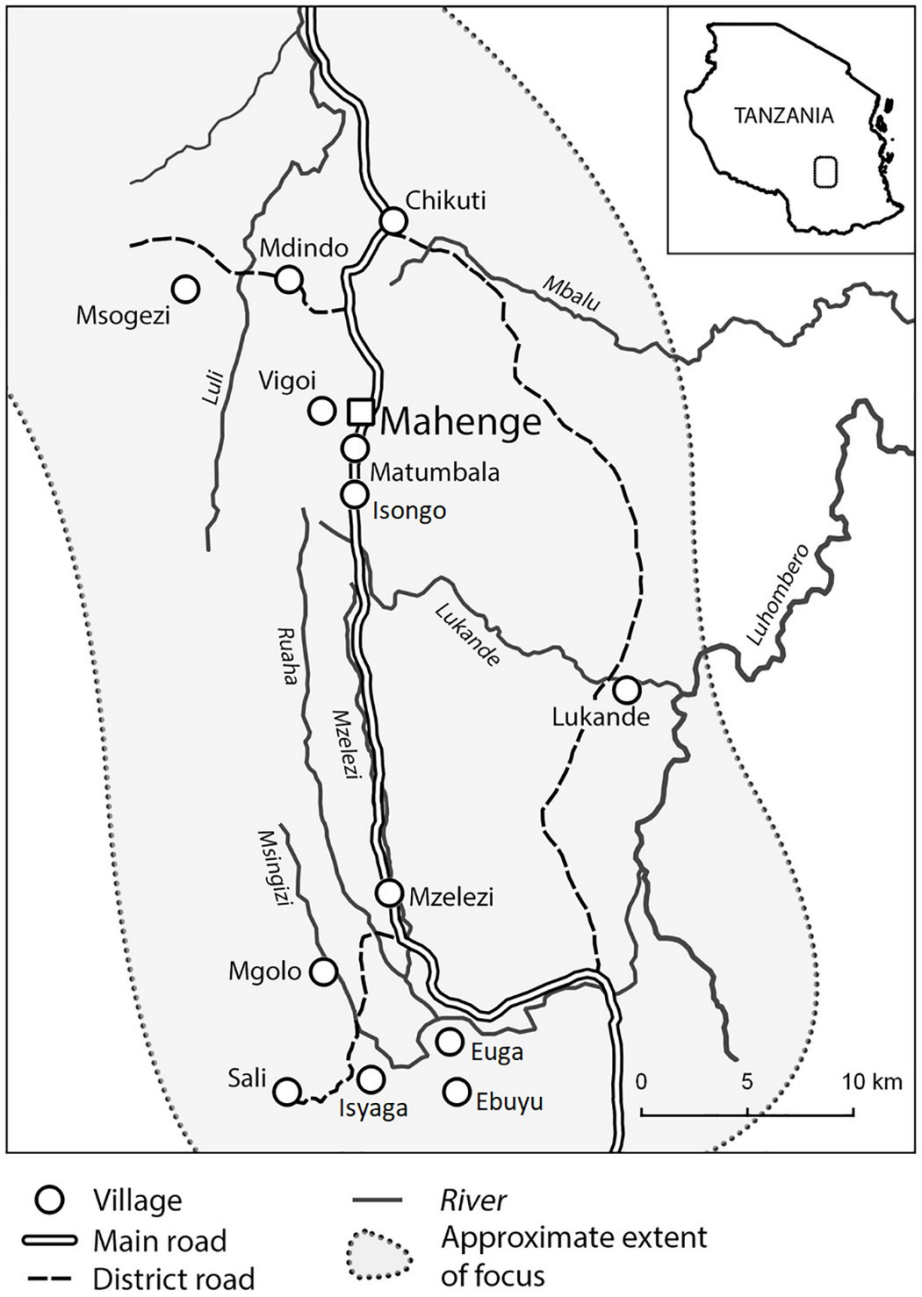
Map of the four study sites, Mdindo, Msogezi, Mzelezi and Sali, in the Mahenge area [16].

A detailed description of the study area and protocol have been published elsewhere [18]. Briefly, the Mahenge area (Fig 1), located in the Morogoro region of south-eastern Tanzania, is among the most onchocerciasis-endemic foci in this country. The area has mountains where fast-flowing rivers originate, providing breeding sites for blackflies. Mahenge is home to the Wapogoro population, which depends on farming, livestock keeping (chicken, goats and pigs, the latter mainly kept in suburban villages in small, elevated huts) and gemstone mining.

### 2.3 Study design

A door-to-door epilepsy survey was conducted before implementing bi-annual CDTi in four rural villages in Mahenge: Mdindo and Msogezi in January 2017 and Mzelezi and Sali in February 2018. In August 2021, these door-to-door epilepsy surveys were repeated with a similar methodology in all four villages after implementing bi-annual CDTi since 2019.

Screening for epilepsy was done using a two-step methodology. First, during the door-to-door household visits, trained community health workers collected sociodemographic information about each household (e.g. age, sex and size of the family) and identified every household member with a unique identification number. Then, the community health workers administered a questionnaire to all family members. Our questionnaire (S1 File) was based on a five-question epilepsy screening survey validated in Mauritania [19] and subsequently extensively used in epilepsy studies in resource-limited settings, particularly in sub-Saharan Africa [20], and also included a question regarding the ivermectin intake of each household member in the year preceding each survey. This questionnaire was discussed in detail during a training session with the community workers and translated into the local language, Kiswahili, using terminologies that were well understood and agreed upon by all community workers. In the second step, suspected individuals were invited to a central point to be interviewed and examined by a trained medical doctor (DB) to confirm or reject the epilepsy diagnosis (S2 File). All confirmed PWE were treated with antiseizure medication.

In June 2022, community health workers providing antiseizure medication in the four villages were asked about new cases of epilepsy in the community. Moreover, PWE in the four study villages were interviewed with a standardised questionnaire to determine when they developed their first seizures.

### 2.4 Definition and classification of epilepsy

Epilepsy was defined as two or more unprovoked seizures at least 24 hours apart [21]. A person with OAE was defined as a previously healthy person living in an onchocerciasis-endemic area for at least three years who had developed epilepsy without an obvious cause between the ages of 3 and 18 years [3]. Nodding seizures were defined as epileptic seizures with the head repeatedly dropping forward. A probable NS (pNS) case was defined according to the modified WHO case definition established in 2012 [22].

### 2.5 Incidence of epilepsy

PWE identified during the pre- and post-intervention surveys were asked the age at which they had their first seizures. The annual incidence of overall epilepsy and of pNS was then determined by subtracting the age of seizure onset from the current age of the PWE. This information was harmonised and cross-checked between the datasets using the unique identification number given to each participant.

### 2.6 Assessment of CDTi coverage

All household members were asked whether they took ivermectin during the CDTi round in 2016 in Mdindo and Msogezi and the two CDTi rounds in 2021 (March and August 2021) in the four villages. For children below 12 years, this information was obtained from the parents/caretakers. New onset cases of epilepsy between 2019 and 2021 were asked about their past ivermectin intake in the 2022 questionnaire.

### 2.7 Ov16 seroprevalence among children aged six to 10 years

All children aged six to 10 years in the study villages were invited to undergo an Ov16 IgG4 antibody test as an indicator of recent exposure to *O*. *volvulus* [2]. In the 2017/18 pre-intervention surveys, whole blood from a finger prick was used for Ov16 rapid diagnostic testing (RDT) (SD Bioline, Inc, Gyeonggi-do, South Korea) in the field. In 2021, dry blood spots were obtained from finger pricks and later, after dried blood spot elution, tested in the laboratory using the same Ov16 RDT.

### 2.8 Data analysis

The sociodemographic characteristics of the participants were described as number (n) and percentage frequency (%) for categorical variables and median and interquartile range (IQR) for continuous data. The overall epilepsy prevalence was calculated by dividing the confirmed cases of epilepsy before (in January 2017 for Mdindo and Msogezi and February 2018 for Mzelezi and Sali) and after (in 2021 for all villages) implementing bi-annual CDTi by the total number of people screened in each of those years. The overall epilepsy incidence was estimated for the three years prior to each survey (in 2015–2017 for Mdindo and Msogezi and 2016–2018 for Mzelezi and Sali, and 2019–2021 for all villages) by dividing the number of new cases by the respective summed person-years of the population at risk, as follows:

$$\text{Incidence}=\frac{\text{New}\;\text{epilepsy}\;\text{cases}\;\text{in}\;\text{the}\;\text{three}\;\text{years}\;\text{before}\;\text{the}\;\text{survey}}{\text{Total}\;\text{population}\;\text{surveyed}\;\text{x}\;\text{3}}$$

Prevalence and incidence of pNS were calculated the same way as overall epilepsy. Ninety-five per cent confidence intervals (95%CI) were presented for prevalence and incidence estimates using Wilson Score Intervals with Yate’s continuity to account for rare events [23]. The death and migration of community members during the three years of the study were assumed to have a minimal effect on the incidence and prevalence. Overall epilepsy and pNS incidence and onchocerciasis seroprevalence among six to 10 years old children were compared between pre- and post-intervention. Ivermectin coverage was defined as the proportion of persons who reported taking ivermectin over the total population in the survey. Mann–Whitney U test was used to explore differences between the two time periods (before and after intervention) in continuous variables.

## 3. Results

### 3.1 Characteristics of the study population

The number of people screened was 5,444 in 2017/18 and 6,598 in 2021, of which 3,261 (49.4%) were males and 3,336 (50.6%) were females in 2021 (sex information was not available for one participant). The median age of the population was 18.1 (IQR: 9.2–36.1) years in 2021. During the study period, the population size in all villages increased except in Mdindo. There were no significant differences in the proportion of females and age distributions between villages and time periods.

### 3.2 Prevalence of epilepsy

The overall prevalence of epilepsy was comparable in 2017/18 and 2021 (Table 1), respectively, 33.4 (95%CI: 28.9–38.6) and 30.5 (95%CI: 26.5–35.0) per 1,000 people. Similarly, the prevalence of pNS was 6.6 (95%CI: 4.7–9.3) in 2017/18 and 6.2 (95%CI: 4.5–8.5) in 2021 per 1,000 people. The median age of the epilepsy cases was 24.4 (IQR: 18.4–35.0) in 2017/18 and 27.0 (IQR: 18.8–37.0) in 2021 (p = 0.18). Epilepsy prevalence was similar in females and males (in 2017/18: 3.6% females versus 3.0% males, p = 0.25; in 2021: 3.3% females versus 2.9% males, p = 0.329).

**Table 1:** Prevalence of overall epilepsy and of probable nodding syndrome in four rural villages in Mahenge pre- and post-implementing bi-annual CDTi intervention.

Villages	Pre-intervention (2017/18 surveys)					Post-intervention (2021 survey)				
	Participants screened n (%)	PWE		pNS		Participants screened n (%)	PWE		pNS	
		n	n/1000 (95%CI)	n	n/1000 (95%CI)		n	n/1000 (95%CI)	n	n/1000 (95%CI)
**Mdindo**	941 (14.3)	33	35.1 (24.6–49.5)	5	5.3 (2.0–13.1)	849 (12.9)	36	42.4 (30.3–58.8)	6	7.1 (2.9–16.1)
**Msogezi**	1,558 (23.6)	55	35.3 (26.9–46.0)	13	8.3 (4.6–14.6)	2,001 (30.3)	60	30.0 (23.1–38.7)	13	6.5 (3.6–11.4)
**Mzelezi**	1,769 (26.8)	51	28.8 (21.8–38.0)	13	7.4 (4.1–12.9)	2,312 (35.0)	58	25.1 (19.3–32.5)	16	6.9 (4.1–11.5)
**Sali**	1,176 (17.8)	43	36.6 (26.9–49.4)	5	4.3 (1.6–10.5)	1,435 (21.8)	47	32.8 (24.4–43.7)	6	4.2 (1.7–9.6)
**Total**	5,444 (100.0)	182	33.4 (28.9–38.6)	36	6.6 (4.7–9.3)	6,597 (100.0)	201	30.5 (26.5–35.0)	41	6.2 (4.5–8.5)

n–Number; PWE–Persons with epilepsy; pNS–Probable nodding syndrome; CI–Confidence interval.

During the 2021 survey, 26 new cases of epilepsy that had not been reported in the 2017/18 surveys were identified. Of those, nine (34.6%) developed their first seizures after 2018. The remaining 17 (63.0%) reported an onset of epilepsy before 2017 and therefore were missed in the 2017/18 surveys, five in Mdindo, four in Msogezi, four in Mzelezi and four in Sali.

### 3.3 Overall incidence of epilepsy and pNS pre- and post-implementing bi-annual CDTi

Between 2015/16-2017/18 and 2018–2021, there was a significant decline in the incidence of overall epilepsy from 177.6 (95%CI: 121.2–258.5) to 45.5 (95%CI: 22.2–89.7) per 100,000 person-years and a non-significant reduction in the incidence of pNS from 18.4 (95%CI: 4.7–58.5) to 5.1 (95%CI: 0.3–32.8) per 100,000 person-years (Table 2).

**Table 2:** Incidence of overall epilepsy and of probable nodding syndrome pre- and post-implementing bi-annual CDTi in the four rural villages.

Surveys	Pre-intervention incidence (2015/16-2017/18)* (data obtained during the 2017/18 surveys)*				Post-intervention incidence (2019–2021) (data obtained during the 2021 survey)			
	Overall epilepsy incidence		pNS incidence		Overall epilepsy incidence		pNS incidence	
	n	n/100,000 (95%CI) PY	n	n/100,000 (95%CI) PY	n	n/100,000 (95%CI) PY	n	n/100,000 (95%CI) PY
**Mdindo**	4	141.7 (45.4–389.0)	1	35.4 (1.9–229.8)	0	0.0 (0.0–187.8)	0	0.0 (0.0–187.8)
**Msogezi**	8	171.2 (79.6–351.5)	1	21.4 (1.1–138.8)	2	33.3 (5.8–134.3)	0	0.0 (0.0–79.7)
**Mzelezi**	12	226.1 (122.6–406.8)	1	18.8 (1.0–122.3)	6	86.5 (35.2–198.5)	1	14.4 (0.8–93.6)
**Sali**	5	141.7 (52.2–351.1)	0	0.0 (0.0–111.2)	1	23.2 (1.2–150.7)	0	0.0 (0.0–111.2)
**Overall**	29	177.6 (121.2–258.5)	3	18.4 (4.7–58.5)	9	45.5 (22.2–89.7)	1	5.1 (0.3–32.8)

n–Number; pNS–Probable nodding syndrome; CI–Confidence Interval; PY–Person-years.

* Mdindo and Msogezi were surveyed in 2017 (incidence between 2015–2017), and Mzelezi and Sali were surveyed in 2018 (incidence between 2016–2018)

Of the nine new epilepsy cases in 2019–2021, six met the criteria of OAE (Table 3). Eight of the new cases did not take ivermectin in the year they developed seizures. Of these, six did not take ivermectin before they experienced their first seizures (cases 2–4, 7 and 8), and three took ivermectin before but skipped treatment the year they developed seizures (cases 1, 5 and 9). The sixth case, in which ivermectin intake information was not available, was not an OAE case but a confirmed case of severe malaria.

**Table 3:** Characteristics of the nine new persons with epilepsy onset in 2019–2021.

New Epilepsy cases	Village	Sex	Age	Obvious cause of epilepsy	Age of first seizures	pNS	Met OAE criteria	Took ivermectin in the year of the first seizures
**1**	Msogezi	Female	6	No	5	No	Yes	No
**2**	Mzelezi	Female	12	No	11	Yes	Yes	No
**3**	Mzelezi	Female	15	No	14	No	Yes	No
**4**	Mzelezi	Female	25	No	23	No	No	No
**5**	Msogezi	Male	8	No	7	No	Yes	No
**6**	Mzelezi	Male	7	Severe malaria (3 years ago)	5	No	No	Not known
**7**	Mzelezi	Male	13	No	12	No	Yes	No
**8**	Mzelezi	Male	26	No	25	No	No	No
**9**	Sali	Male	18	No	18	No	Yes	No

pNS–Probable nodding syndrome; OAE–Onchocerciasis-associated epilepsy.

No new cases of epilepsy, including NS, were identified by the community workers since the house-to-house survey in August 2021. In June 2022, none of the PWE interviewed in the four villages reported that their seizures started after October 2021.

### 3.4 CDTi coverage

In 2016, the annual CDTi in Mdindo and Msogezi villages achieved coverage of 79.8% (95%CI: 78.5–81.0%) in individuals aged above six years. Bi-annual CDTi has been implemented since 2019. However, in 2020, there was only one ivermectin distribution due to the COVID-19 pandemic.

During the biannual CDTi in 2021, 82.3% of the population at risk took ivermectin at least once (Table 4), and the coverage in individuals aged above six years was 90.4% (95%CI: 89.6–91.2%). The first round of CDTi, in March 2021, achieved a higher coverage (p < 0.0001) and had no significant difference between the four villages. However, in the second round, in August 2021, the coverage was significantly lower in Sali (70.5%, 95%CI: 68.0–72.9%). The CDTi coverage was higher in children and teenagers aged six to 18 years (93.2%, 95%CI: 92.1–94.2%) than in adults (89.5%, 95%CI: 88.4–90.5%).

**Table 4:** Biannual CDTi coverage in 2021 overall and per round.

Village		Mdindo	Msogezi	Mzelezi	Sali	Overall
**CDTi coverage**^A^ n/T % (95%CI)	**1**^**st**^ **round** (March 2021)	619/793 78.1 (75.0–80.9)	1564/1926 81.2 (79.4–82.9)	1843/2233 82.1 (80.4–83.7)	1129/1371 82.4 (80.2–84.3)	5146/6314 81.5 (80.5–82.5)
	**2**^**nd**^ **round** (August 2021)	615/792 77.7 (74.6–80.5)	1488/1925 77.3 (75.3–79.1)	1779/2224 80.0 (78.3–81.6)	964/1367 70.5 (68.0–72.9)	4846/6308 76.8 (75.8–77.9)
	**Total**^B^ (2021)	632/796 79.4 (76.4–82.1)	1585/1932 82.0 (80.2–83.7)	1854/2228 83.2 (81.6–84.7)	1137/1372 82.9 (80.7–84.8)	5208/6328 82.3 (81.3–83.2)

^A^ Two hundred and seventy people did not provide information about their ivermectin intake and were excluded from the population to calculate the CDTi coverage.

^B^ Persons who took ivermectin in one or both CDTi rounds in 2021.

CDTi–Community-directed treatment with ivermectin; CI–Confidence Interval; n–Number of people who took ivermectin; T–Total number of people with information regarding ivermectin intake.

Most people who did not take ivermectin in 2021 were under five years of age (758 in March, 64.9%; and 760 in August, 52.0%) or pregnant (64 pregnant women in March, 5.5%; 49 pregnant women in August, 3.4%). Thirty-four out of 81 (42.0%) pregnant women in 2021 took ivermectin in one of the CDTi rounds that year, before or after pregnancy.

### 3.5 Ov16 seroprevalence in children

Ov16 seropositivity among children aged six to 10 years was 23.6% (95%CI: 20.2–27.5%) in 2017/18 and 28.2% (95%CI: 24.7–32.0%) in 2021 (Table 5).

**Table 5:** CDTi coverage in 2021 and Ov16 seroprevalence among children aged six to 10 years pre- and post-strengthening control interventions in the four rural villages.

Village		Mdindo	Msogezi	Mzelezi	Sali	Overall
**Ov16 seropositive** n/T % (95%CI)	**2017/18** ^A^	31/91 34.1 (24.7–44.8)	68/167 40.7 (33.2–48.6)	9/178 5.0 (2.5–9.6)	21/110 19.1 (12.5–27.9)	129/546 23.6 (20.2–27.5)
	**2021** ^B^	32/115 27.8 (20.1–37.1)	41/159 25.8 (19.3–33.4)	27/152 17.8 (12.2–25.0)	73/187 39.0 (32.1–46.5)	173/613 28.2 (24.7–32.0)

^A^ Rapid diagnostic test was conducted in the field.

^B^ Rapid diagnostic test was conducted in the laboratory.

CI–Confidence Interval; n–Number of children Ov16 seropositive; T–Total number of children tested for Ov16 seropositivity.

## 4. Discussion

This study aimed to determine the impact of switching from an annual to a bi-annual CDTi programme on the incidence of epilepsy, including NS, in four rural villages with high epilepsy and onchocerciasis prevalence in the Mahenge area. With the implementation of the bi-annual CDTi since 2019, the incidence of epilepsy decreased from 177.6 (121.2–258.5) to 45.5 (95%CI: 22.2–89.7) per 100,000 person-years. Six (66.7%) of the 2021 epilepsy incident cases met the criteria of OAE. Moreover, no new epilepsy cases were reported between August 2021 and June 2022. These results confirm the previously reported decrease in epilepsy incidence by onchocerciasis elimination efforts in other onchocerciasis-endemic areas with a high epilepsy prevalence [9, 10, 24]. Similar to Mahenge, most PWE had OAE in these other onchocerciasis-endemic areas, and thus interrupting *O*. *volvulus* transmission decreased the incidence of epilepsy, including NS.

In Cameroon, two cohort studies showed that the risk of developing epilepsy increased gradually with high *O*. *volvulus* microfilarial densities during childhood [25]. Ivermectin has an embryostatic effect on adult worms that suppresses microfilariae production. This effect gradually decreases and ceases after a few months, and microfilarial production resumes [4]. Hence, treating the population with bi-annual CDTi will not only contribute to the elimination of onchocerciasis transmission but also protect children over five years of age from high microfilarial loads during the entire year, halting their risk of developing OAE. Lowering the minimal age for ivermectin treatment to three could make children free of high microfilarial loads at a younger age and consequently further reduce their risk of developing OAE. However, ongoing clinical trials need to determine the safety, optimal dose, and formulation of ivermectin for treating children below five years of age [26].

There was no significant decrease in epilepsy prevalence between 2017/18 (3.3%, 95%CI: 2.9–3.9%) and 2021 (3.1%, 95%CI: 2.7–3.5%). As epilepsy is a chronic condition, its prevalence can only decrease if PWE die [27]. Because antiseizure medication had been distributed to all identified PWE since 2019, epilepsy-related mortality was limited over the three years between surveys. We observed that the median age of PWE shifted by about three years (from 24.4 to 27.0), albeit non-significantly. With a longer follow-up, a decrease in epilepsy prevalence and a shift in the median age of PWE to an older population are expected. This was the case in Northern Uganda [9]. Another study also found that, after the elimination of onchocerciasis in western Uganda, the overall epilepsy prevalence between 1994 and 2018 decreased from 29.9 (95%CI: 21.3–41.9) to 11.6 (95%CI: 7.8–17.1) per 1,000 people [10].

The non-significant change in the Ov16 seroprevalence among children aged six to 10 years between 2017/18 and 2021 was to be expected, given the short timeframe of this study. The high Ov16 prevalence in children reveals the previous high intensity of transmission of *O*. *volvulus*, which is related to OAE incidence [28]. Children in 2021 likely already had Ov16 antibodies before implementing the bi-annual CDTi. Moreover, it is also possible that the sensitivity of the Ov16 RDT to detect Ov16 antibodies is slightly higher when the test is performed in the laboratory compared to the field.

The population increased in all villages except Mdindo, reflected in the slight increase in the total number of PWE in the area. The population decreased in Mdindo, most likely due to resettlement to other locations because of the planned mining. The epilepsy prevalence in Mdindo did not decline, most likely as a result of the emigration of healthy household members, while PWE remained in the village to obtain antiseizure treatment.

A bi-annual CDTi programme was implemented in Mahenge with high coverage of 82.3% (95%CI: 81.3–83.2%) in 2021, as recommended to accelerate onchocerciasis transmission interruption [13]. This coverage was significantly higher than the one in 2016 (p < 0.0001), which was still delivered annually. Moreover, the high biannual coverage in 2021 was sustained in each of the two CDTi rounds (81.5% and 76.8%), with nearly all persons eligible for CDTi taking ivermectin (>90%), especially children and teenagers (93%). The latter may result from an OAE awareness campaign explaining that onchocerciasis might induce OAE and that this form of epilepsy can be prevented by taking ivermectin [29]. Still, none of the six new onsets of OAE cases took ivermectin in the year they developed their first seizures. This shows the importance of providing ivermectin to all eligible children and teenagers to prevent OAE.

Our study has several limitations. First, the pre- and post-bi-annual CDTi populations were not completely the same. Population movements between and outside the study area may have impacted the outputs obtained.

A second limitation is that epilepsy incidence information was obtained by asking PWE identified during the pre- and post-intervention surveys when they experienced their first seizures. This information may have been subject to recall bias.

A third limitation was the small sample size. There was a non-significant decrease in the incidence of pNS after implementing bi-annual CDTi, which could have been a significant reduction if the statistical power of the study had been larger.

A fourth limitation is that we did not have an external control group. Indeed, it was considered not ethical to include a control group with only annual CDTi because bi-annual CDTi is recommended by WHO for high onchocerciasis transmission areas. Therefore, we cannot exclude that an unrecognised confounding factor could have decreased the incidence of epilepsy. SARS-CoV-2 pandemic preventive measures were not implemented in Tanzania, and therefore no major change in everyday life was observed during the study period.

Another limitation is that no CDTi coverage surveys were performed in 2019 and 2021 and only one round of CDTi happened in 2020 due to the COVID-19 pandemic. However, nationally reported CDTi coverage from the area did not reveal differences in the CDTi coverage of 2019, 2020 and 2021 data. In 2021, we detected that several cases of epilepsy in the 2017/18 surveys were missed, most likely because of epilepsy-associated stigma and reluctance to disclose the epilepsy condition [30]. We cannot exclude that in 2021 additional cases were missed. However, the latter is unlikely, as a community-based epilepsy treatment programme was established in the study villages in 2019, and interventions have been implemented to reduce epilepsy stigma, such as explaining the association between onchocerciasis and epilepsy and epilepsy peer support groups [31].

A final limitation of this study is the different way the Ov16 RDT was performed during the two surveys (RDT field test in 2017 and RDT performed in the laboratory in 2021).

## Conclusion

Introducing a bi-annual CDTi programme in Mahenge decreased the incidence of epilepsy in the rural study villages. Another population-based study with a larger sample size should be conducted to confirm the Mahenge findings by evaluating the effect of biannual CDTi in an onchocerciasis-endemic area of high epilepsy incidence. Ideally, a surveillance system should be set up in such a study to identify new cases of epilepsy as they emerge. In the meantime, based on the Mahenge experience, a bi-annual CDTi programme with high coverage is recommended in areas with high prevalence of onchocerciasis and epilepsy. High, uninterrupted CDTi coverage among children is particularly important to prevent OAE. Such a program should also ensure early epilepsy diagnosis and treatment and an uninterrupted supply of antiseizure medication to decrease epilepsy-related mortality and morbidity.

## Supporting information

S1 File Household screening questionnaire. The household screening questionnaire was given to all household elements in 2017 and 2021. This is the questionnaire of 2017.
(DOCX)

S2 File Neurological questionnaire. The neurological questionnaire was administered to the suspected cases of epilepsy by trained medical doctors in 2017 and 2021 to confirm or reject the epilepsy diagnosis. This is the questionnaire of 2017.
(DOCX)

S1 Data Household and Epilepsy dataset of 2021. (XLSX)

S2 Data Epilepsy dataset of 2017–2018. (XLSX)

S3 Data Ov16 seropositivity among children aged 6 to 10 year in 2017–2018 and 2021. (XLSX)
