# Supplementary material for: Impact of a bi-annual community-directed treatment with ivermectin programme on the incidence of epilepsy in an onchocerciasis-endemic area of Mahenge, Tanzania: A population-based prospective study
Source: PLoS Negl Trop Dis. 2023 Jun 28;17(6):e0011178. doi: 10.1371/journal.pntd.0011178 (PMC10335697; doi:10.1371/journal.pntd.0011178)
Supplement: S1 File — The household screening questionnaire was given to all household elements in 2017 and 2021. This is the questionnaire of 2017. (DOCX) [file pntd.0011178.s001.docx]

S1 File — Household screening questionnaire.

*NOTE: This questionnaire will be administered in a digitalized form on a tablet computer that will be used as a data collection tool during this survey. Within each household, all participants will be asked all the questions in sections 1.2 and 1.3 during one single interview sequence.*

DATE: _____ / _____ / ________
FULL NAME OF INTERVIEWER: _______________________________________________

HEALTH ZONE: _______________________________________________
HEALTH AREA: _______________________________________________

VILLAGE: _______________________________________________

**1.1. HOUSEHOLD CHARACTERISTICS**

UNIQUE HOUSEHOLD CODE:
 XX /XX /XX/XXXX (First 2 letters of: District/Parish/Village, followed by number (e.g.0001))

GPS coordinates of household:
LATITUDE __ __. __ __ __ __ __LONGITUDE __ __. __ __ __ __ __ ALTITUDE _ __ __ __

Full name of household head: _______________________________________________
Mobile phone number of household head: ___________________________________________
Ethnic group of household head: _______________________________________________
Total number of people in the household: ___________________________________________

Main income generating activity of the family:  Farmer  Livestock breeder  Fisherman  Employee
  Professional  Craftsman  other, specify:____________________

Has there been a family member that died from any form of epilepsy?
 YES NO DON’T KNOW

IF YES: When (year) ______ At what age: _____ years

IF YES: When (year) ______ At what age: _____ years

**1.2. INDIVUDUAL INTERVIEW WITH EACH HOUSEHOLD MEMBER**

Person ID: _______________________________________________
Full Name: _______________________________________________
Age: ___ years
Birth date: ___/___/____
Sex: Male Female

Is the person present during the interview visit? YES NO
Who is answering to the questions? self mother household head other, specify _______________

**1.3. SCREENING FOR EPILEPSY CASES**

*If at least one of the 5 questions is answered with YES, the electronic questionnaire will automatically report the person for invitation to participate in the neurological survey for case verification.*

**QUESTION 1.**Have you ever lost consciousness and experienced:

1. Loss of bladder control? YES NO DON’T KNOW

b) Foam at the mouth? YES NO DON’T KNOW

**QUESTION 2.**Have you ever experienced absence(s) or sudden loss(es) of contact with the surroundings, for a short duration of time?

YES NO DON’T KNOW

**QUESTION 3.**Have you ever experienced sudden, uncontrollable twitching or shaking of your arms, legs or head, for a period of a few minutes?

YES NO DON’T KNOW

**QUESTION 4.**
Do you sometimes experience sudden and brief bodily sensations, see or hear things that are not there, or smell strange odours?

YES NO DON’T KNOW

**QUESTION 5.**Have you ever been told that you are suffering from epilepsy or that you have already had epileptic fits?

YES NO DON’T KNOW

**1.3. IVERMECTIN USE**

Have you been taking ivermectin during the last distribution in May 2016?

YES NO DON’T KNOW
