# Supplementary material for: Impact of a bi-annual community-directed treatment with ivermectin programme on the incidence of epilepsy in an onchocerciasis-endemic area of Mahenge, Tanzania: A population-based prospective study
Source: PLoS Negl Trop Dis. 2023 Jun 28;17(6):e0011178. doi: 10.1371/journal.pntd.0011178 (PMC10335697; doi:10.1371/journal.pntd.0011178)
Supplement: S2 File — The neurological questionnaire was administered to the suspected cases of epilepsy by trained medical doctors in 2017 and 2021 to confirm or reject the epilepsy diagnosis. This is the questionnaire of 2017. (DOCX) [file pntd.0011178.s002.docx]

S2 File — Neurological questionnaire.

DATE: _____ / _____ / ________

FULL NAME OF THE DOCTOR: _______________________________________________

**PARTICIPANT IDENTIFICATION**

Participant ID _______________________________________________

Last name *(in capital letters)*: _______________________________________________

First name: _______________________________________________

*The medical doctor/neurologist will first assess whether the person referred to him has epilepsy (Nodding syndrome or another form of epilepsy)*

*Diagnosis of Nodding syndrome or another form of epilepsy is confirmed*

YES NO

If NO: other diagnosis ?

Recurrent febrile convulsions

Dizziness / syncope

Paroxysmal vertigo

Severe anaemia

Mental retardation without epilepsy
  Psychiatric illness without epilepsy

Classic migraine

Other, specify______________________

*If YES the following neurology questionnaire will be administered in a digitalized from using tablet computers. By indicating the individual Participant ID on paper forms, the neurologist can provide additional important notes of the anamnesis on paper.*

**PARTICIPANT IDENTIFICATION**

Participant ID _______________________________________________

Last name *(in capital letters)*: _______________________________________________

First name: _______________________________________________

Address *(any information that allows locating the individual)*:

_______________________________________________

_______________________________________________

Town / Village: _______________________________________________

District: _______________________________________________

Phone number: _______________________________________________

Sex: Male Female

Age: _______________
Date of birth: ___/___/____

Place of birth: _______________________________________________

Country of birth: _______________________________________________

Ethnic group: _______________________________________________

What is the participant’s birth order among his / her siblings? First born second third more than third

Marital status: Married Living with parents In partnership
 Living alone other, specify ____________________

Is the participant answering himself / herself?  YES  NO

If NO, who is answering and what is the relationship between the participant and respondent?
Full name: _______________________________________________

Relation to participant: mother father sibling other, specify _______________

**DEMOGRAPHIC DATA**

What is the interviewed participant's duration of stay in the survey area? ________ years
  Transit  less than 1 year

Since birth  DON’T KNOW

IF less than 1 year: how many MONTH?: __ __

IF less than 1 year, in which village has the participant lived before? Village: ___________________________________

Area: ___________________________________
 Health zone: ___________________________________

IF in a foreign country: in which country has the participant lived before? _______________________________________________

Are you currently attending school?  YES NO

School grade level completed: None
 Primary  P1 P2 P3 P4 P5 P6 P7

Secondary  S1 S2 W3 S4 S5 S6

Tertiary  University  Vocational college/Institute

Certificate of primary education:  YES NO

IF NO: if the participant had stopped school attendance:
 at which level? ____________________________

Reason:  Due to epileptic seizures while in school
 Fear to leave the epileptic child unattended
 Due to epilepsy related stigma
 Due to epilepsy related learning difficulties

other reasons (financial, other illness than epilepsy, accessibility,)

DON’T KNOW

ADULT Participants:

Occupation / main activity:  Housekeeper  Farmer  Livestock breeder  Fisherman  Employee  Craftsman  Student  Professional  None  other, specify:_________________________________

CHILD Participants:

Occupation / main activity of family:  Housekeeper  Farmer  Livestock breeder  Fisherman  Employee  Craftsman  Student  Professional  None  other, specify:_________________________________

**HISTORY OF EPILEPSY**

***SEIZURE TYPOLOGY***

Has the participant ever lost consciousness and experienced:

1. Loss of bladder control? YES NO DON’T KNOW

c) Foam at the mouth? YES NO DON’T KNOW

1. Biting of the tongue? YES NO DON’T KNOW

Has the participant ever experienced absence(s) or sudden loss(es) of contact with the surroundings, for a short duration of time? YES NO DON’T KNOW

Has the participant ever experienced sudden, uncontrollable twitching or shaking of your arms, legs or head, for a period of a few minutes? YES NO DON’T KNOW

Does the participant sometimes experience sudden and brief bodily sensations, see or hear things that are not there, or smell strange odours? YES NO DON’T KNOW

Has the participant ever been told that he / she is suffering from epilepsy or that he / she has had epileptic fits? YES NO DON’T KNOW

Of what type are the most frequent seizures?

Generalized tonic-clonic seizures
  Generalized myoclonic seizures
  Atonic seizures (drop attacks)
  Absences
  Simple partial (focal) seizures (consciousness not lost)
  Complex partial (focal) seizures (decreased consciousness)
  Secondarily generalized partial seizures
  Others, specify:_____________________________

Does the participant have a history of head nodding?
  YES, in the past  YES, still ongoing

NO  DON’T KNOW

IF YES, specify age of the participant at onset of head nodding ______ years

What triggers the seizures / head nodding? (tick all that apply)

Spontaneous (no obvious trigger)
 Sight of food Cold weather

Nothing DON’T KNOW

Other, specify _____________________________

Aura before fits:  YES  NO  DON’T KNOW

***SEIZURE HISTORY***

What is the number of epileptic seizures since onset?

One seizure  Two or more seizures

If only two seizures, were they more than 24h apart?  YES  NO  DON’T KNOW

Has the participant had a seizure in the last 5 years?

YES  NO  DON’T KNOW

Has the participant had a seizure in the last 12 month?

YES  NO  DON’T KNOW

IF YES, in which month has the last seizure been experienced?
 MONTH: _ _  DON’T KNOW

What is the current frequency of the seizures?

yearly (if less than 1 per month)

monthly (if less than 4 per month)

weekly (if less than 7 per week)

daily (if more than 7 per week)

Specify number:_____________per ______________

How many seizures did you have LAST WEEK?

number____  None  DON’T KNOW

Was the onset of seizures within the first year of life?  YES  NO  DON’T KNOW

If the onset of seizures was after the age of one, at what age? ____ year  DON’T KNOW

***MEDICAL HISTORY***

Family history of seizures  YES  NO  DON’T KNOW

IF YES, specify who these are (tick all that apply)

Siblings (brother/sister); No. of affected siblings ______
 Father Mother Grandparent(s)

Does the participant have a twin?  YES  NO  DON’T KNOW

IF YES does the twin have seizures?  YES  NO  DON’T KNOW

Family history of other mental illness?

YES  NO  DON’T KNOW

IF YES, specify disorder _____________________________________________

***Pregnancy and Birth:***

Is the mother of the participant present for this part of the interview?  YES  NO

Did the pregnancy of the mother of the participant proceed normally?

YES  NO  DON’T KNOW
If NO, specify: ___________________________________

Was the participant born at term (pregnancy had completed 9 months)?

YES  NO  DON’T KNOW

Mode of delivery for the participant:  Spontaneous Vaginal Delivery
  Assisted Vaginal Delivery (forceps, suction, etc.)

Caesarean section

Birthplace of the interviewed participant?
 DON’T KNOW
 At home
 Health centre
 Dispensary
 Hospital
 Other, specify:

Did the participant cry immediately?  YES  NO  DON’T KNOW

What was the birth weight? ______________________GRAMS  DON’T KNOW

***Psychomotor Development during Childhood:***

**Prior to onset of seizures**

Was the child growing normally prior to the onset of the seizures?
  Yes  No  DON’T KNOW

IF NO, at what age did the abnormal growing appear? ______ years

Did the child learn to do things like other children of his/her age prior to the onset of the seizures?  Yes  No  DON’T KNOW

IF NO, at what age did the learning difficulty start? ______ year

Compared with other children of his/her age, did the child appear in any way mentally backward, dull or slow before the onset of the seizures?  Yes  No  DON’T KNOW

IF YES, at what age did it start? ______ years

**Since the onset of seizures**

Compared with other children of his/her age, did the child learned to do things like other children?

Normal  Delayed  Abnormal
  Others, specify:_____________________________

Compared with other children of his/her age, did the child appeared in any way mentally backward, dull or slow?

Normal  Delayed  Abnormal
  Others, specify:_____________________________

***Occurrence of severe disease in the past:***

Has the interviewed participant suffered from severe measles preceding the onset of epileptic seizures?
  YES  NO  DON’T KNOW

Has the interviewed participant suffered from a severe form of malaria preceding the onset of epileptic seizures?
  YES  NO  DON’T KNOW

Has the interviewed participant suffered from encephalitis/meningitis preceding the onset of epileptic seizures?
  YES  NO  DON’T KNOW

Has the participant had a head injury with loss of consciousness preceding the onset of epileptic seizures?  YES  NO  DON’T KNOW

Has the participant had a prolonged posttraumatic coma before the onset of epileptic seizures?
  YES  NO  DON’T KNOW

Was the onset of epilepsy following another illness?
  YES  NO  DON’T KNOW
If YES, specify the illness: _____________________________________________

***TOXICANTS***

Has the participant a history of excessive alcohol consumption?
  YES  NO  DON’T KNOW  Not applicable*

Has the participant a history of drugs abuse?
  YES  NO  DON’T KNOW  Not applicable*

*In case the participant is a young child.

***GENERAL EXAMINATION***

BODY WEIGHT (kg): _________ . ____kg

HEIGHT (cm): _______________cm

How is the general condition of the interviewed participant?

GOOD AVERAGE POOR
 DON’T KNOW

Thoracic abnormalities  YES  NO

IF YES, specify ________________________________________

Facial abnormalities  YES  NO

IF YES, specify ____________________________________________

Does the adolescent (> 16years old) /adult looks like a child?  YES  NO

If yes, external signs of sexual development conform to age:

YES  NO  EXAMINATION DECLINED

If NO, specify: girls:  breast not developed
girls and boys:  no pubic hear

Dermatology  NORMAL  ABNORMAL

Burn scars  YES  NO

Itching  YES  NO

Papular eruption  YES  NO

Depigmented lesions (leopard skin)  YES  NO

Suspected onchocerciasis nodules  YES  NO

***NEUROLOGICAL EXAMINATION***

Is the participant alert?  YES  NO

Fully oriented in place/time/person  YES  NO

Is the participant’s mentally retarded?  YES  NO

Ophthalmology  NORMAL  ABNORMAL VISION
  BLIND, BOTH EYES AFFECTED

Normal eye movements?  YES  NO

Normal hearing  YES  NO

Generalised muscle wasting  YES  NO

Paresis  YES  NO

if YES specify__________________________________________________________

Contractures  YES  NO  DON’T KNOW

Is the participant walking normally?  YES  NO  DON’T KNOW

IF NO, specify  Ataxic (wide base) gait

Waddling gait (like a duck)

Spastic gait –with tip toe walking

Hemiplegic – with one sided weakness

Other, specify______________________

***Psychiatric symptoms***

Does the participant has hallucinations, i.e. sees, hears, smells, feels or tastes things that don’t exist?

YES  NO

Does the participant have delusions, i.e. strongly held false belief by participant despite superior evidence against belief?  YES  NO

Does the participant show aggressive episodes?

YES  NO

Have you felt very sad (irritable) for a period of more than two weeks?  YES  NO

Have you experienced loss of interest and pleasure in almost all activities for a period of more than two weeks?  YES  NO

In the past month, have you been having strong memories / dreams of something bad that happened to you or your loved one?  YES  NO

Does to participant suffer from another neuro-psychiatric / psychological problem?
  YES  NO

IF YES, specify: __________________________________________

**Physical / Functional Indices**

*Modified Rankin Scale: Please mark the most accurate description of the current functional state of the child, as observed during the evaluation*

| **Score** | **Description** |
| --- | --- |
| 0 | No symptoms at all |
| 1 | No significant disability despite symptoms; able to carry out all usual duties and activities |
| 2 | Slight disability; unable to carry out all previous activities, but able to look after own affairs without assistance |
| 3 | Moderate disability; requiring some help, but able to walk without assistance |
| 4 | Moderately severe disability; unable to walk without assistance and unable to attend to own bodily needs without assistance |
| 5 | Severe disability; bedridden, incontinent and requiring constant nursing care and attention |
| 6 | Dead |
| **SCORE (0-6): ________** | |

Neurological exam  NORMAL  ABNORMAL

***CASE CLASSIFICATION***  Epilepsy
  Head nodding syndrome

Head nodding syndrome plus

***ANTI-EPILEPTIC TREATMENT***

What is or was the type of seizure medication taken by the participant?

No treatment  DON’T KNOW
  Traditional  anti-epileptic drug
  Mixed

*If anti-epileptic drug treatment:* Which substance is taken by the participant?

Barbiturates  YES  NO  DON’T KNOW
Sodium valproate  YES  NO  DON’T KNOW
Diphenylhydantoin  YES  NO  DON’T KNOW
Carbamazepine  YES  NO  DON’T KNOW
Other anti-epileptic treatment  YES  NO  DON’T KNOW
If YES, specify: _____________________________________________

*Compliance*: Is the participant taking the treatment regularly?

YES  NO  DON’T KNOW

If NO, why?  Personal reasons
  (Temporary) non-availability of medication
  Lack of financial means to buy medication
  DON’T KNOW
  Other, specify ________________________________

**IVERMECTIN USE**

Has the participant ever received ivermectin?  YES  NO  DON’T KNOW

NOT APPLICABLE (according to exclusion criteria, as follows):

1) age <5 years at the moment of CDTi; 2) pregnancy; 3) Breast feeding < 7 days; 4) acute severe disease: 1 2 3 4

IF YES: Has the participant taken ivermectin during the last CDTi in May 2016

YES  NO  DON’T KNOW

How many times per year?  ONCE  TWICE  DON’T KNOW

IF NOT taken in 2016, why?  no distribution  absent during the CDTi  refused
  afraid of secondary effects  pregnancy
  breastfeeding an infant younger than 7days
  because I was asked to NOT take it
  age <5 years at the time of CDTi
  severe acute disease at the time of CDTi
  other, specify _______________________________

IF TAKEN in 2016, why?  for its beneficiary effects.
  it is recommended to be taken
  to decrease itching
  other, specify _______________________________

Was Ivermectin/Mectizan distributed in another way than orally?

YES NO DON’T KNOW

Is the participant followed up for epilepsy treatment?

YES  NO  DON’T KNOW

If yes, where?

Mahenge epilepsy treatment center

Other, specify _________________________________

Does the participant allows to consult the medical information at the epilepsy treatment centre

YES  NO

OV16 rapid test done:  YES  NO

IF YES: OV16 RDT result:  POSITIVE  NEGATIVE

Picture or video taken:  YES  NO

**Action Taken by reporting officer**:

Participant referred to treatment centre  YES  NO

Other action, specify: ____________________________________________________________
